# Detection of Pulmonary tuberculosis: comparing MR imaging with HRCT

**DOI:** 10.1186/1471-2334-11-243

**Published:** 2011-09-16

**Authors:** Elisa Busi Rizzi, Vincenzo Schinina', Massimo Cristofaro, Delia Goletti, Fabrizio Palmieri, Nazario Bevilacqua, Francesco N Lauria, Enrico Girardi, Corrado Bibbolino

**Affiliations:** 1 Diagnostic Department, Radiology. "L. Spallanzani" National Institute for Infectious Diseases Rome ITALY; 2 Research Department, Translational Research Unit. "L. Spallanzani" National Institute for Infectious Diseases Rome ITALY; 3 Clinical Department. National Institute for Infectious Diseases "L. Spallanzani" Rome; 4 Epidemiology Department, "L. Spallanzani" National Institute for Infectious Diseases Rome ITALY

## Abstract

**Background:**

Computer Tomography (CT) is considered the gold standard for assessing the morphological changes of lung parenchyma. Although novel CT techniques have substantially decreased the radiation dose, radiation exposure is still high. Magnetic Resonance Imaging (MRI) has been established as a radiation- free alternative to CT for several lung diseases, but its role in infectious diseases still needs to be explored further. Therefore, the purpose of our study was to compare MRI with high resolution CT (HRCT) for assessing pulmonary tuberculosis.

**Methods:**

50 patients with culture-proven pulmonary tuberculosis underwent chest HRCT as the standard of reference and were evaluated by MRI within 24 h after HRCT. Altogether we performed 60 CT and MRI examinations, because 10 patients were also examined by CT and MRI at follow- up. Pulmonary abnormalities, their characteristics, location and distribution were analyzed by two readers who were blinded to the HRCT results.

**Results:**

Artifacts did not interfere with the diagnostic value of MRI. Both HRCT and MRI correctly diagnosed pulmonary tuberculosis and identified pulmonary abnormalities in all patients. There were no significant differences between the two techniques in terms of identifying the location and distribution of the lung lesions, though the higher resolution of MRI did allow for better identification of parenchymal dishomogeneity, caseosis, and pleural or nodal involvement.

**Conclusion:**

Technical developments and the refinement of pulse sequences have improved the quality and speed of MRI. Our data indicate that in terms of identifying lung lesions in non-AIDS patients with non- miliary pulmonary tuberculosis, MRI achieves diagnostic performances comparable to those obtained by HRCT but with better and more rapid identification of pulmonary tissue abnormalities due to the excellent contrast resolution.

## Background

CT is considered the gold standard for assessing the morphological changes of lung parenchyma. Although novel CT techniques have substantially decreased the radiation dose, radiation exposure is still high. Magnetic Resonance Imaging (MRI) has been established as a radiation- free alternative to CT for several lung diseases, explaining the growing interest in (MRI) for lung parenchyma. New technologies and strategies which allow for very fast imaging and improved image quality [1,2] have been introduced, but their role in infectious diseases still needs to be explored further.

MRI of the lung is difficult for several reasons. Major problems result from susceptibility artifacts caused by extensive air-tissue parenchymal interfaces and the low-proton density of normal parenchyma, both of which are factors that lead to low signal intensity of the normal lung. Another problem is the continuous motion of all components induced by heart pulsation and respiration, which are most prominent in the lower and anterior sections of the chest. However, proton density increases when lung tissue damage determines air space obliteration, reducing the susceptibility effects. In these cases, MRI plays a role in assessing lung parenchyma [1,3-5] and could be useful in diagnosing pneumonia, due to the exudative accumulation of water and cells occurring in the air space.

There is some existing information on MR imaging of the lung for various diseases, including pulmonary perfusion and ventilation [1,2,5-9], however to our knowledge, no systematic study has been published about diagnostic MRI in patients with pulmonary tuberculosis.

The aim of our study was to compare HRCT and MRI lung examinations to identify the features of tuberculosis (TB).

## Methods

### Patients enrolment and characteristics

Our prospective study received institutional review board approval (INMI 3/150207) by the local ethical committee and all patients provided written informed consent.

The 50 patients (17 women and 33 men, ranging from 21-63 years of age with a median age of 47) who met our study criteria were referred to undergo HRCT and MRI imaging. The entry criteria for patients were as follows: (a) a chest X-ray with pulmonary abnormalities, (b) culture-proven pulmonary tuberculosis in culture from sputum (also induced) or bronchoalveolar lavage (c) absence of contraindication to MR examinations (ex. cardiac pacemakers, cochlear implants), (d) MRI obtained within 24 hours of the CT examination, to avoid divergent results during therapy.

All patients who did not have AIDS or additional concomitant infectious diseases were undergoing TB treatment. Altogether we performed 60 CT and MRI examinations, because 10 patients were also examined by CT and MRI at follow- up. However we analyzed the latter examinations to increment the number in our series, not to evaluate the effectiveness of therapy

### CT

Low dose HRCT was performed on all subjects using a helical four-channel MDCT scanner (Light Speed QX/i General Electric Medical System, Milwaukee, Wis). Unenhanced HRCT was obtained from the apex to the base of the lung at the end of inspiration, with 1-mm collimation, a high resolution algorithm, and 10 mm spacing. A specific mediastinum reconstruction algorithm was employed, and the images were obtained on lung and mediastinal settings.

To minimize the radiation required for obtaining diagnostic scans, the following scanning parameters were selected: tube current 70 mA, with 100 kV [7].

### MRI

Parallel imaging MRI was performed with a 1, 5-T system (Signa Excite, General Electric Medical System, Milwaukee, Wis, USA), a maximum gradient strength of 33 mT/m, and a slew rate of 120 mT/m/s, using a six-channel body phased array coil system.

The examinations were performed with expiratory respiratory and diastolic gating. When pulsation was less vigorous, pulsation artifacts were reduced [1,6,8]. In agreement with the literature data [6], we preferred to use respiratory gating which allowed for continuous breathing instead of multiple breath hold acquisitions, also because the shifts of the lung parenchyma relative to the slice level are reduced. We performed MRI in expiration because the expiration phase is longer than the inspiration phase and signal intensity increases with deflation [8]. Even when MRI was performed in expiratory respiration and CT at the end of inspiration, there was no significant discrepancy between the breathing positions of the images.

An axial T2-weighted Fast Recovery Fast Spin-Echo (FR FSE T2) FAT SAT was used with the following parameters: Echo Time, 90 msec; Repetition Time, 2500 msec; Echo Train Length, 14; bandwidth, 50; slice thickness, 5 mm; slice gap, 2 mm; field of view, 42 cm; matrix size, 288 × 224, reconstructed to 512.

Fat saturation sequences are very effective because the attenuated fat signal of the thoracic subcutaneous tissue reduces the ghosting artifacts of the ventral chest wall [6] and also increases conspicuity of fluids [9].

This sequence provides good image quality, and with imaging times of about 120 seconds we obtained enough slices to assess the entire lung.

The in-room time, including positioning the patient on the examination table, was approximately 15 minutes.

### Imaging Analysis

Both CT and MR images, all made anonymous, were directly displayed on the monitors of a picture archiving and communication system (PACS 5.1 Kodak, Rochester, NY, USA) with a window setting appropriate for lung parenchyma and mediastinum (pixel 2048 × 2560, display gradation 1021 (10-bit), maximum brightness 750cd/m^2^, LCD display device 54 cm). The readers were asked to assess presence, location and extension of pulmonary TB.

According to the standardized nomenclature for parenchymal findings on CT, consolidation was defined as a homogenous increase in lung parenchyma attenuation that obscures the margins of the vessels and airway walls (an air bronchogram may be present); a nodule was defined as a round lesion with a diameter of 3 cm or smaller; ground glass pattern was defined as a homogenous, hazy area of increased attenuation without obscuration of bronchovascular margins (an air bronchogram may be present); cavitation was defined as a gas-filled space, contained or not contained within a pulmonary consolidation, mass, or nodule. Tree in bud appearance was defined as a linear branching structure with more than one contiguous branching site. Furthermore, we assessed interstitial changes, in particular miliary, bronchial wall and peribronchial tissue thickening. Pleural and mediastinal lymph node involvement was also assessed.

Pleural effusion was defined as free-flowing pleural fluid producing sickle-shaped opacity (in most cases posteriorly) and loculated fluid collections as lenticular opacities in a fixed position. Pleural effusions with a volume of 15 ml or more can be detected with CT, however pleuritis sicca is not visible on CT scans. Lymph nodes were considered enlarged when they were greater than one centimeter on the short axis. Since there are no established MRI criteria to define parenchymal lung findings, we adopted the CT criteria. Regarding the pleura, MRI can detect subtle signal abnormalities that might be consistent pleuritis sicca. Concerning lymph nodes, we also assessed nodal signal intensity compared with thoracic wall muscle. Previous reports correlated histological data with MRI features in tuberculous lymphadenopathy [10], including signal intensity on unenhanced MR. Based on MRI findings, lymph node types could be defined according to the presence and degree of granuloma formation, caseation/liquefaction necrosis, fibrosis and calcifications. Signal intensity may differ depending on the stage of evolution: i) slight hyper-intensity may reflect lymphoid hyperplasia related to inflammation, ii) high hyper-intensity is suggestive of liquefactive necrosis, and iii) central isointensity associated with peripheral hyper-intensity may reflect caseosis.

The MRI findings to be assessed were previously established by consensus to avoid bias in individual interpretation.

All MR images were independently analyzed by two board-certified radiologists (VS, EBR, both with 10 years of experience in clinical MR imaging and 25 years of experience in chest imaging). The observers were unaware of CT results to avoid interpretation bias. Since CT is considered the gold standard technique, all CT images were considered as reference scans and were analyzed in a randomised order by the same radiologist in consensus, two months after analyzing the MR images.

Then, they directly compared MRI with CT examinations in consensus to verify the presence, distribution and characteristics of pathological features. In divergent cases, MRI and CT were re-examined to determine which imaging technique was correct. Disagreements in image scorings were resolved by consensus. MRI artifacts were graded as minimal (barely visible), moderate (clearly visible, but not interfering with evaluation) and severe (compromising evaluation). Particular attention was given to determining whether these artifacts interfered with the diagnostic value.

For CT and MR images, each parenchymal finding was scored on a scoring sheet using the following sliding scale of relative certainty: 0 = definitely negative; 1 = probably negative; 2 = indeterminate; 3 = probably positive; 4 = definitely positive. To calculate the MRI detection rate for each finding, we only considered those that were scored as 0 (definitely negative) and 4 (definitely positive).

Furthermore, for both imaging techniques, we classified each lung by zone: upper, middle, and lower, resulting in a total of six zones per patient. The upper zones were defined as areas of the lung above the level of the carina; the middle zones as areas between the level of the carina and the origin of the inferior pulmonary veins; and the lower zones as areas below the origin of the inferior pulmonary veins. Each zone had approximately the same number of sections. We scored lung zone involvement by using a four-point scale: 0 = no involvement, 1 = < 25%, 2 = 25%-50%, 3 = > 50%.

### Statistical analysis

Statistical analyses were performed using the SPSS/PC+ version 11 (SPSS, Chicago, Ill). A p value lower than 0.05 indicated a statistically significant difference.

The degree of agreement between observers interpreting chest MRI was determined by using pair-wise kappa statistics as follows: very good, k value > 0.81; good, k value 0.80-061; moderate, k value 0.60-041. A separate kappa value was calculated for each sign that was reviewed.

K statistics were also calculated to analyze the agreement between MRI and CT and a detection rate for each finding; in this analysis, results were dichotomized as definitely positive or not.

The chi- square test was used to compare the proportion of images demonstrating different scores of involvement for each zone of the lungs depicted by MRI and CT.

## Results

MR artifacts were negligible in 48 cases (48/60 80%), and minimal (barely visible) in 12 cases (12/60 20%), however, these artifacts did not interfere with the diagnostic value. There were no motion artifacts or instances of image distortion due to susceptibility that resulted in poor image quality.

Pathological findings were observed in all CT and MR examinations (Table 1) however, in our study we did not observe miliary or calcified parenchymal lesions.

**Table 1:** Findings of 60 HRCT examinations and 60 MRI examinations

	HRCT score	MRI score				
		0	1	2	3	4
Consolidation	52					52
Alteration consistent with caseosis	undetermined					4
Cavitation	39					39
Ground glass	6			2	1	4
Nodule	43					43
Tree in bud	33					29
Interstitial changes	14			1	4	13
Lymph nodes	(Short axis > 10 mm) 38					56
Edema	undetermined					50
Alteration consistent with colliquative necrosis	undetermined					2
Alteration consistent with caseosis	undetermined					4
Pleural involvement	10					21

0 = definitely negative

1 = probably negative

2 = indeterminate

3 = probably positive

4 = definitely positive

Concerning MR imaging, agreement between observers was always very good for each sign (k = 0.98-0.86).

**Figure 1:**
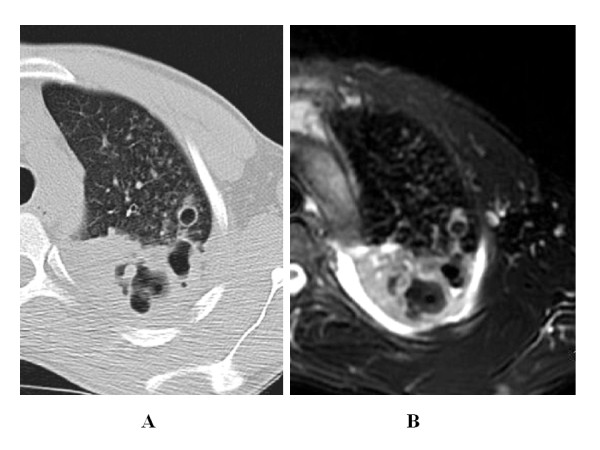
**32 year-old man with pulmonary tuberculosis**. A) HRCT shows parenchymal consolidation with cavitations. B) Unenhanced MRI better depicts caseosis, air level in the cavities and pleural effusion.

A k value of 1 for MRI/CT agreement was recorded for consolidations, nodules and cavities. In 4 patients, MRI easily assessed caseation while CT showed aspecific hypodensity (Figure 1a, b).

**Figure 2:**
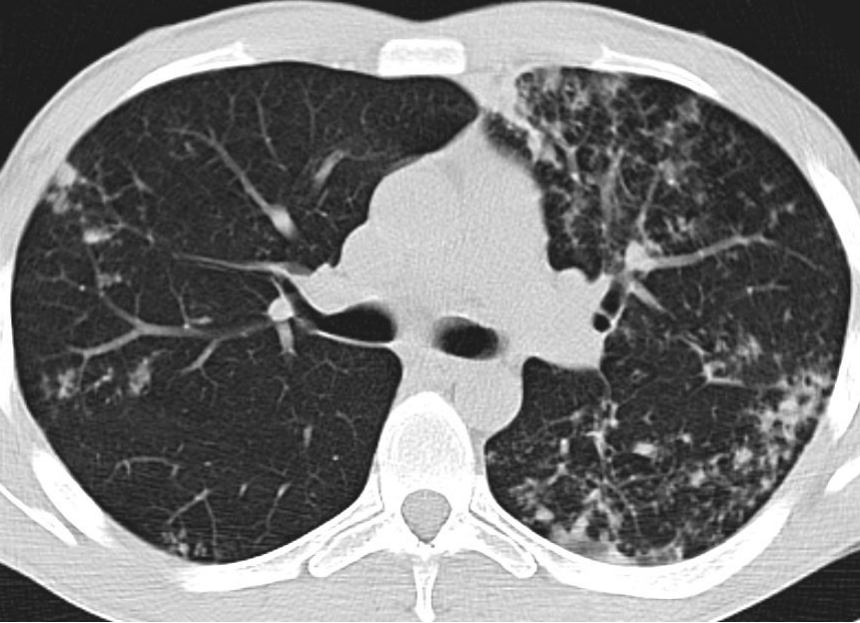
**28 year-old man with pulmonary tuberculosis, HRCT shows bronchogenic spread and interstitial changes with peribronchial thickening**.

**Figure 3:**
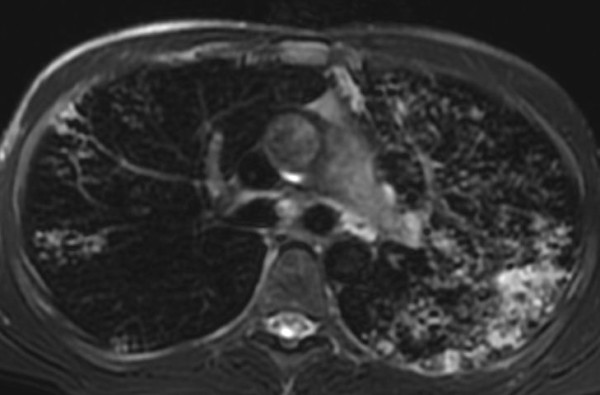
**Same patient reported in Fig.2: 28 year-old man with pulmonary tuberculosis, unenhanced MRI identifies the same features as well as HRCT in figure 2**.

**Figure 4:**
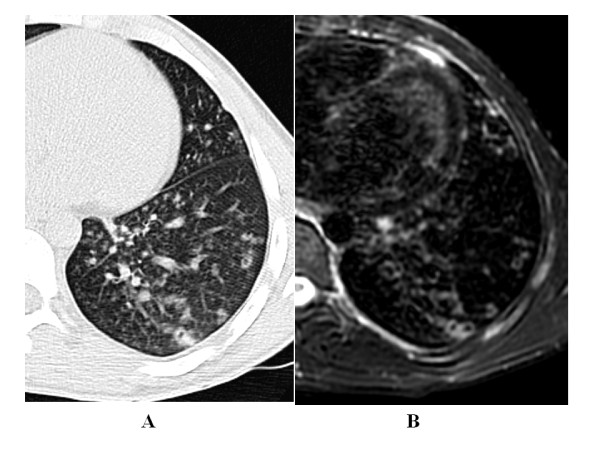
**44 year-old man with pulmonary tuberculosis**. A) HRCT shows interstitial changes with peribronchial thickening. B) Unenhanced MRI identifies the same features as well as HRCT.

**Figure 5:**
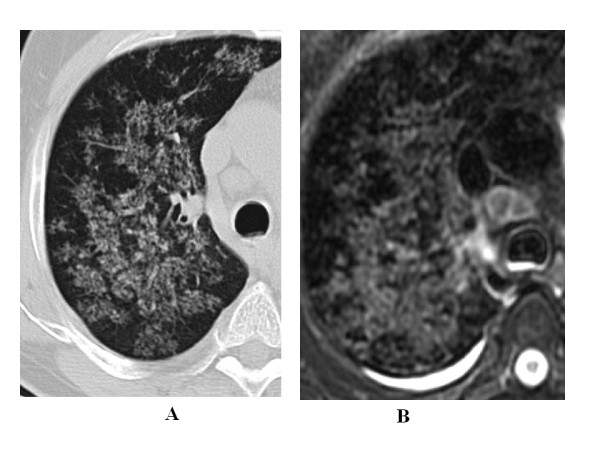
**53 year-old woman with pulmonary tuberculosis**. A) HRCT shows interstitial changes with peribronchiolar thickening. B) Unenhanced MRI identifies the same features as well as HRCT and also depicts lymph node caseosis.

The k value for MRI/CT agreement was 0.90 for ground glass, 0.86 for tree in bud and 0.78 for interstitial changes (Figure 2, Figure 3, Figure 4a, b, Figure 5a,b).

**Figure 6:**
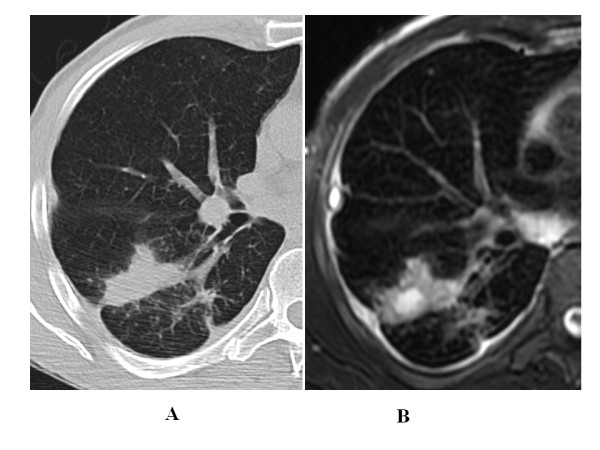
**38 year-old man with pulmonary tuberculosis**. A) HRCT shows parenchymal consolidation. B) Unenhanced MRI depicts colliquative necrosis within consolidation, subtle, free and loculated pleural effusion, and a highly hyper-intense mediastinal lymph node.

**Figure 7:**
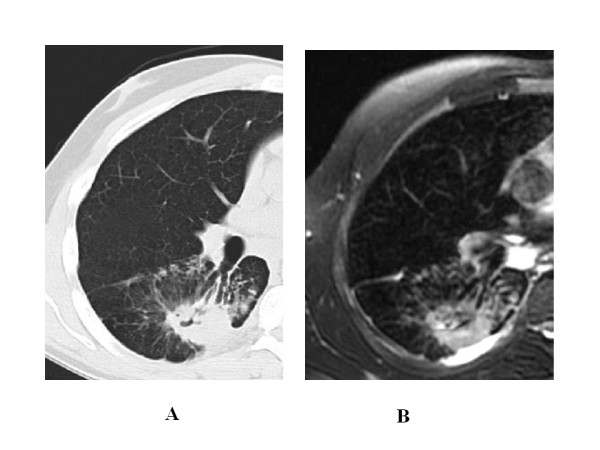
**61 year-old woman with pulmonary tuberculosis**. A) HRCT shows cavited infiltrate. B) Unenhanced MRI identifies the same features as well as HRCT and identifies subtle, free pleural effusion, and a highly hyper-intense mediastinal lymph node.

Regarding pleural effusion (Figure 6a, b, Figure 7a, b), the results of the two techniques differed considerably, and the k value for MRI/CT agreement was 0.54. For MRI, we found hyper-intensity to be consistent with pleural involvement in 35% cases (21/60 examinations). For CT, pleural effusion (free or loculated) was seen in only 17% (10/60 examinations) of the cases. In the remaining 11 cases depicted by MRI, 3 cases showed very subtle pleural effusion (identified on CT by the reviewers in consensus), and in 8 there was no pleural abnormality on CT; pleural layers showed a relatively high signal intensity on T2-weighted images without significative thickening or effusion, consistent with inflammation sicca.

**Figure 8:**
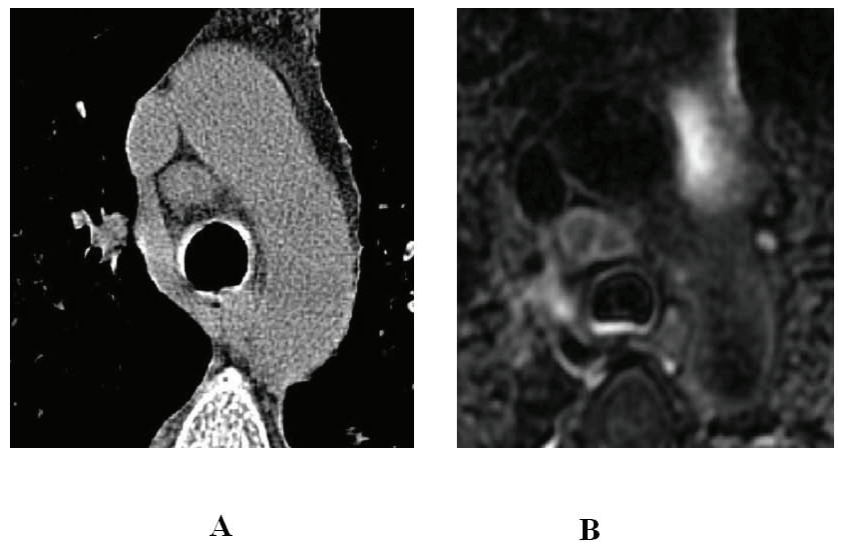
**53 year-old woman with pulmonary tuberculosis**. A) HRCT, reconstructed for mediastinum, shows an enlarged pretracheal lymph node, B) Unenhanced MRI also depicts lymph node caseosis.

Regarding mediastinal lymph nodes (Figure 8a, b), on MRI we found that in 23% of the cases (14/60 examinations) there was either enlargement and/or signal alteration consistent with nodal involvement; a total of 56 lymph nodes were evaluated. In our series, when compared with the thoracic wall muscle, 89% of the nodes were slightly hyper-intense (most likely reflecting lymphoid hyperplasia), 7% showed central isointensity associated with peripheral hyperintensity (most likely reflecting caseosis), and 3.5% were highly hyper-intense, suggesting liquefactive necrosis.

A total of 38 lymph nodes were evaluated on unenhanced CT. Nodal involvement, assessable only as an enlargement, was seen in 16% of the cases (10/60 examinations). In the remaining 4 patients, nodal involvement (depicted by MRI as hyperintensity) was not identified on CT by the reviewers in consensus, nor at re-examination, because the nodal short axis remained < 10 mm.

We examined the lymph nodes (which were identified as pathological on HRCT and MRI because the short axis was greater than 10 mm) to choose which imaging technique enabled correct diagnosis of nodal involvement. We observed that MRI depicted signal alterations in all of the lymph nodes that were enlarged on CT. The same MR signal alterations were observed in lymph nodes that were not enlarged on CT. These data suggest that MRI have a higher sensitivity for detecting nodal involvement. In all patients, both MR and CT examinations showed identical results concerning the location of parenchymal features (Table 2).

**Table 2:** Detection and percentage of pulmonary involvement in 60 HRTC and 60 MRI images

Location	Score 0		Score 1		Score 2		Score 3		P value
	CT	MRI	CT	MRI	CT	MRI	CT	MRI	
Right lung									
Upper zone	224	224	33	33	3	3	32	24	0.79 (ns)
Middle zone	48	48	33	24	40	24	16	16	0.40 (ns)
Lower zone	265	249	40	57	8	9	9	10	0.31 (ns)
Left lung									
Upper zone	116	116	40	40	48	48	182	182	1 (ns)
Middle zone	24	24	24	24	32	24	74	83	0.64 (ns)
Lower zone	99	88	107	116	40	32	16	25	0.27 (ns)

0 = no involvement

1 = < 25%,

2 = 25%-50%,

3 = > 50%.

## Discussion

Today CT represents the gold standard for assessing lung parenchyma. MRI has some relevant clinical application, but it is not used in routine clinical management. Several recent studies clearly demonstrated that MRI can identify lung abnormalities [1,2,4-6,9] with significant diagnostic advantages over CT in terms of higher contrast resolution and absence of exposure to radiation. However, CT is cheaper, more widespread, rapid to perform, and has an established role in clinical management. On the other hand, the disadvantages of MRI, such as limited spatial resolution and motion and susceptibility artifacts, have been overcome by using new techniques. In agreement with literature, our study indicates that regarding presence and distribution of pathological lung features, MRI and CT show the same results, even regarding lung tuberculosis.

Based on a one- to -one correlation between MRI and CT, the findings in both techniques regarding consolidations, nodules and cavities correlated well, and indeed obtained the same results when identifying these characteristics.

On the contrary, although MRI identified parenchymal changes, on 4 occasions it failed to diagnose "tree in bud", probably misinterpreted as interstitial changes. These false negative results for bronchogenic spread were made because of the lower MR spatial resolution. Likewise, the missed identification of interstitial changes, verified in one follow- up case, was due to very subtle involvement and lower MR spatial resolution.

Moreover small areas of "ground glass" were missed 3 times, misinterpreted as blurring.

It might be useful to consider performing MRI at the end of inspiration, or using a FR FSE T2 without fat sat to visualize the parenchyma [7,11,12] in order to reduce these limiting factors.

However, because of the excellent contrast resolution, MR examinations show immediate results and are even more accurate in revealing lymph node involvement, pleural abnormalities and parenchymal caseation than unenhanced CT.

Indeed, MRI indicated nodal involvement in 14 patients and parenchymal caseation in 4 of these, features not clearly identified by the CT. However, the CT examination was unenhanced, thus could not allow for correct diagnosis. To our knowledge, no investigators have focused on the MRI features of thoracic tuberculous lympadenopathies. In our series, 89% were slight hyper-intense, 7% were isointense with peripheral hyper-intensity, and 3.5% were highly hyper-intense compared with the thoracic wall muscle. Signal intensity may differ depending on the stage of evolution, where slight hyper-intensity may indicate lymphoid hyperplasia related to inflammation, high hyper-intensity is suggestive of liquefactive necrosis, and central isointensity associated with peripheral hyper-intensity may indicate caseosis. All these findings are in line with previous reports regarding abdominal tuberculous lymphadenopathy that correlated histological data with MRI features [13].

Because of its excellent contrast resolution, MRI is superior to CT in assessing pleural involvement [14]. Indeed, MRI promptly depicted pleural abnormalities with a higher percentage than CT (35% vs. 17%) with the same immediateness for subtle or loculated effusions (not seen on CT) and pleural hyper-intensity, consistent with early sicca inflammation, which was never observed on CT.

From our experience, MR was not only useful for integrating diagnostic evaluation of the lung, but the results of our study even suggest that MRI could replace CT in assessing lung tuberculosis in some subsets of patients such as children, women, during pregnancy (MR imaging should be avoided during the first trimester), follow -ups, or as an alternative to CT for patients with allergies to IV contrast material. Performing MRI in children with TB may also prove to be interesting since they may have lymph node involvement rather than lung involvement; however it should be taken into account that anaesthesia may be required.

## Conclusion

We believe that MRI is comparable to CT for identifying morphological pulmonary changes, and that MRI is clearly superior to CT regarding tissue characterization. Furthermore, on the basis of lesion signal intensity, MRI could differentiate the exudative stage of lung TB from the relatively acellular fibrotic phase because of the relatively "short T2" in fibrotic tissues [15].

The shortcomings of our study are the limited number of patients and the absence of miliary and calcified nodules found in the enrolled patients, thus it could not be proved that these lesions can be identified by MRI. The lack of histopathological correlation or microbiological tests of the adenopathies is another important limitation of the study, and MRI in the phase of expiration may show non-pathological processes such as small laminar atelectasis. Moreover, further studies are needed to determine whether the use of FR FSE T2 sequences without fat sat might improve visualization of the parenchyma.

## Competing interests

The authors declare that they have no competing interests.

## Authors' contributions

EBR and VS have made substantial contributions to the conception and design, acquisition, analysis and interpretation of data, and also drafted the manuscript; MC carried out the radiological examination; EG was responsible for the statistical analysis; DG enrolled the patients and drafted the manuscript; FP, NB were responsible for patient selection and enrolment; DG, EG, FNL, CB revised the manuscript critically for important intellectual content.

All authors have read and approved the final manuscript.

## Pre-publication history

The pre-publication history for this paper can be accessed here:

http://www.biomedcentral.com/1471-2334/11/243/prepub
